# How Does Corporate Social Responsibility Affect Sustainability of Social Enterprises in Korea?

**DOI:** 10.3389/fpsyg.2022.859170

**Published:** 2022-03-02

**Authors:** Chenglin Qing, Shanyue Jin

**Affiliations:** ^1^Department of Business Administration, Honam University, Gwangju, South Korea; ^2^College of Business, Gachon University, Seongnam, South Korea

**Keywords:** SEs, corporate social responsibility, sustainability, performance, innovativeness, South Korea

## Abstract

Social enterprises (SEs) are a new concept, integrating corporate profitability and social purposes. SEs seek to realize sustainable social values, rather than short-term profits. It is therefore important to study the factors that affect the sustainable management of SEs. Corporate social responsibility (CSR) is known to improve corporate image and performance; it can also promote the sustainable development of companies. Innovation has been described as the driving force behind corporate growth and ultimate performance. This study aims to investigate whether CSR can affect sustainability through the economic and social performance of SEs. In addition, it attempts to verify the moderating role of innovativeness in the relationship between CSR and social enterprise (SE) performance. Using survey data from 226 employees of 204 SEs in Korea, we have empirically tested this conceptual framework. The results suggest that, while CSR can improve sustainability through economic and social performance, innovativeness has no moderating effect on the relationship between CSR and SE performance. This study enriches our understanding of the important role played by CSR in driving SE sustainability. It provides new insights into the mechanisms through which SEs can achieve sustainable development. It also contributes to the literature by emphasizing the need for innovation through technical support for SEs.

## Introduction

Social enterprises (SEs) are organizations that produce, sell, and promote products and services, while supporting social causes (Steiner and Teasdale, 2019). They fall between non-profit and profit-making enterprises (Kerlin et al., 2021). While the main goal of a traditional business model is to maximize shareholder interests, SEs are different because they help to create jobs and develop local communities, including vulnerable groups, with social problem-solving as a top priority (Cheah et al., 2019). After the International Monetary Fund crisis in the 1990s, various academic opinions emerged in South Korea regarding the effectiveness of government support. Although government support expanded social service-related jobs, they were neither stable nor long-term. In this context, South Korea is introducing SEs to help develop local communities and the national economy by providing high-quality social services, while supporting social and economic values.

Social enterprises that pursue both societal and economic values increase their social influence through corporate social responsibility (CSR). An organization undertakes CSR activities to advance the sustainable development of society, as a responsible member aiming to maximize profits, which is the basic goal of most commercial enterprises (Javed et al., 2020). To solve social problems, SEs strive to attain enhanced societal and economic performance through job creation, community development, and social-service provision simultaneously (Steiner and Teasdale, 2019). Thus, SEs must realize both economic and social values in order to grow sustainably.

Recently, changes in corporate goals have necessitated the development of new business goals (Moon and Parc, 2019). In particular, although enterprises are striving to make social contributions, social distrust in ethical responsibility or CSR has increased, while trust in—and the reputations of—many businesses have declined. This situation has given rise to the view that enterprises should implement economic revitalization through sustainable growth. In the same context, SEs can help to develop society by fostering social and economic value in the social economy (Richter, 2019). It is thus important to strengthen competitiveness by practicing sustainable management through authentic CSR activities.

Social enterprises should aim to achieve both social and economic performance through strengthened competitiveness (Bhattarai et al., 2019). In addition, SEs, which need to become more competitive by overcoming physical and human resource-related issues, should look for more sustainable growth through CSR. In one interpretation, sustainable growth is taking responsibility for the local community by considering the ethical and moral aspects of a business alongside economic growth. Organizations aligned with this approach develop management activities and goals to achieve better long-term outcomes (Bebbington and Gray, 2001; Moizer and Tracey, 2010).

Although, increasingly, SEs need and show interest in CSR, discussions and studies of improved performance and sustainable growth through CSR remain insufficient (Ketprapakorn and Kantabutra, 2019; Powell et al., 2019). Most research has focused on improving enterprise image through CSR and the effect of corporate economic performance in response to stakeholder needs (Abdulaziz-Alhumaidan and Ahmad, 2019; Zhu et al., 2019; Bahta et al., 2021). It is crucial to explore the effect of CSR on two types of performance: social and economic (Cheah et al., 2019). It is also worth investigating the mediating effect of these two types of performance on the relationship between CSR and sustainability (Powell et al., 2019). Accordingly, the present study discusses the importance of CSR and policy measures on sustainable growth in SEs.

This study analyzes the effects of CSR on performance and sustainability by focusing on SEs. Previous studies (Voss and Voss, 2000; Miles et al., 2014) of social enterprise (SE) performance have categorized their results based on economic and social performance. To ensure sustainable growth among SEs that pursue both profit and public interest, our study reveals the relationship between CSR, performance, and sustainability. In addition, creative ideas and product, process, and management innovations are essential elements of SEs, due to their rapid technological convergence and innovation (Pierce and Delbecq, 1977).

Given the increase in public interest in social responsibility and environmental problems in Korea, the number of SEs is continuously increasing. SEs contribute to the regional economy by creating jobs, solving social problems, and developing local communities. However, these enterprises are highly dependent on government subsidies and lack sustainability, which requires innovation. The present study has therefore focused on improving the sustainability of SEs. Social responsibility, selected as a variable to promote sustainability, affects the performance of SEs. In other words, social responsibility activities can improve a company’s performance and ultimately lead to sustainable growth. The present study has analyzed the mediating role of performance in the relationship between CSR and sustainability. In addition, SEs can improve their performance and achieve sustainability through increasing levels of innovative behavior. For this reason, the present study has focused on innovation as a moderating variable.

Therefore, this study aims to investigate whether CSR can affect sustainability through the economic and social performance of SEs. In addition, it attempts to verify the moderating role of innovativeness in the relationship between CSR and SE performance.

This research differs from previous studies. As the CSR of SEs is influenced by performance and sustainable growth, we track and verify the moderating effect of innovation and the mediating effect of performance. Innovation is a moderating variable in the relationship between performance and sustainability. A higher level of innovation will strengthen the effect of performance on sustainability. Finally, this study identifies the CSR levels needed to foster growth in SEs.

## Literature Review and Hypotheses

### Corporate Social Responsibility

As demand for ethical trading has increased, CSR has become increasingly important. CSR has a positive impact on enterprise performance; it is also an essential factor in sustainable growth, regardless of the type or size of the enterprise in question (Gürlek et al., 2017). SEs that provide jobs and services to socially vulnerable people lead the way in realizing social values through CSR (Cornelius et al., 2008). They prioritize CSR, which ultimately strengthens local-community capability and integration by pursuing high autonomy and continuous profit-making activities. This approach improves the quality of life of local residents, minimizes societal problems and potential issues caused by enterprise activities, and maximizes social contributions to meet the needs of stakeholders and society as a whole (Pomering and Johnson, 2009).

Corporate social responsibility is an organization’s responsibility for society, beyond economic, and legal obligations (Carroll, 1991). The organization voluntarily undertakes social roles, solves social and environmental problems, and harmonizes with societal values, norms, and expectations (Sethi, 1975). To satisfy all of the obligations associated with enterprise-management activities, the organization must perform economically, legally, ethically, and economically in a way that upholds its social responsibilities (Carroll, 1979). In addition, SEs that view CSR as a top priority use it as a survival strategy, contributing to society through social influence (Aras et al., 2010) and using social influence to meet their societal responsibility to provide sustainable growth.

Corporate social responsibility has developed into a new form of management strategy, which generates economic benefits associated with sustainable growth and uses environmental and social responsibilities strategically to create a strong, long-term competitive advantage (McAdam and Leonard, 2003). Recently, as sustainable growth has been incorporated into CSR, researchers have recognized that sustainable growth is an indispensable factor in the survival of SEs (Chen and Kelly, 2015). In other words, CSR is an important strategy, which allows SEs to secure an ongoing competitive advantage (Igwe et al., 2018). In the past, organizations pursued CSR to enhance their business image; now, they aim to account for the needs of various stakeholders while practicing CSR from a strategic and sustainable growth perspective (Hong and Chao, 2018).

### Performance

Performance is the achievement and evaluation of organizational outcomes over a certain period of time (Bovaird and Rubienska, 1996). SEs that seek both public interest and profit pursue social goals, involving both social and economic value (Richter, 2019). For such organizations, performance is the degree to which both economic and social values result from management activities carried out over a certain period of time (Bagnoli and Megali, 2011). SE performance incorporates both economic performance (e.g., profits generated through the production and sale of products and services) and social performance, which creates positive values in society, such as job creation, the inclusion of vulnerable groups, environmental conservation, community contributions, and social-service provision (Bhattarai et al., 2019).

Although there is some debate over the measurement index and the best way to measure the performance of SEs, most previous studies have divided the performance of SEs into social and economic performance (Moizer and Tracey, 2010; Miles et al., 2014; Liu et al., 2015; Cheah et al., 2019; Doh, 2020; Pinheiro et al., 2021). Economic performance refers to the generation of profits that enable SEs to operate independently, without government support. Survival and sustainability require both economic performance and profit generation (Leung et al., 2019). Chang and Hong (2000) define economic performance as the level of financial profit creation and economic independence required to manage a company continuously. In other words, the economic performance of a SE is the monetary effect of the sale of its products and services (Bagnoli and Megali, 2011; Doherty et al., 2014).

The social performance of a SE is the result of its contribution, social value, and sense of duty, goals pursued by all SEs (Luke et al., 2013). Although such enterprises must achieve both social and economic performance, they depend on government support (Bae et al., 2018). SE reflects a range of public-interest achievements, including employing vulnerable groups, providing social services, improving the community’s quality of life, and facilitating community integration (Brammer et al., 2006). The present study therefore divides SE performance into social and economic performance.

### Sustainability

Sustainability, in the corporate or organizational sense, means not just pursuing the goals and activities of an organization, but also achieving and developing them in the long term to achieve better outcomes (Bebbington and Gray, 2001). From an organizational perspective, sustainability is the continual management of activities through the social responsibility of staff members; the consideration of ethical issues, such as social service provisions and environmental problem-solving; and the generation of financial revenue. Sustainability of SE raises the question of whether SEs can achieve their original purpose while maintaining social activities—successfully providing sustainable jobs and expanding social services (Leung et al., 2019).

Arena et al. (2015) have introduced the concept of longevity and a comparative perspective to measure the sustainability of SEs. Compared with other types of businesses, SEs have greater potential in the following areas: future employment, social-service provision, support from government and large corporations, and overall growth and competitiveness. A SE becomes sustainable when it can manage its affairs efficiently without government subsidies, simultaneously pursuing both economic and social goals (Bae and Fiet, 2021). This study evaluates the sustainability of SEs by breaking it down into various aspects, including the continuous expansion of employment and increased sales, continued social-service provision, relations with government agencies, and improved competitiveness.

### Innovativeness

Innovation is the creation or invention of new ideas, which are applied to existing processes and operating methods, resulting in new and convergent changes (Hurt et al., 1977; Pierce and Delbecq, 1977; Daft, 1978; Rogers, 1995). Organization staff can use creative approaches to work, enhance competitiveness by applying innovative ideas to products or services, and choose innovation as a strategic plan for sustainable growth. Innovation is an intentional and planned change, which occurs throughout the lifetime of an enterprise, improving performance. As an intangible resource and source of competitive advantage, innovation is essential for enterprise sustainability (Bates and Khasawneh, 2005).

Social enterprises can develop products and services with a low-cost structure by using creative ideas to change and manage organizational components, thereby creating a process that leads to strong social and economic performance. For SEs, it is essential to adapt to rapidly changing environments, manage innovation for sustainable growth, and improve work efficiency. The process of innovation can be divided into management and technology (Damanpour and Evan, 1984; Delmas and Pekovic, 2018; Abbas and Saǧsan, 2019). Daft (1978) has categorized the goals of innovation as product and service goals, market and value, and technology. In cases where it is impossible to predict environmental change, rapid response through flexible, creative, and innovative ideas will help an organization achieve sustainable growth, while improving its performance.

### Relationship Between Corporate Social Responsibility and Performance

Mishra and Suar (2010) have analyzed the impact of CSR on enterprise performance in manufacturing enterprises in India; they show that CSR has a positive impact on financial and non-financial performance. In a study of the relationship between CSR and enterprise outcomes, Igwe et al. (2018) have shown that CSR is responsible for profit creation, social contributions, social innovation, and improved social performance. Moon and Parc (2019) have studied the effect of CSR in Korean businesses on shared value creation and management performance, finding that CSR has a positive effect on social and economic value and enterprise performance. Cho and Lee (2019) have verified that CSR has a positive effect on financial and social performance.

Hernández et al. (2020) have analyzed the effects of CSR on enterprise-management performance by categorizing social, environmental, and economic-responsibility activities. Their findings confirm that CSR economic-responsibility activities affect non-financial performance, while economic and social-responsibility activities affect financial performance. Chen and Kelly (2015) have studied the effects of social entrepreneurship on enterprise CSR and social performance, dividing CSR into community, philanthropic, and environmental responsibilities. CSR has a positive influence on social performance, with strategic implications for the sustainable growth of SEs. Based on previous studies, we therefore propose the following hypotheses:

Hypothesis 1: CSR has a positive influence on social performance.
Hypothesis 2: CSR has a positive influence on economic performance.

### Relationship Between Performance and Sustainability

Leung et al. (2019) have examined the effects of SE performance on sustainability, showing that the stronger the economic and social performance of a SE, the greater its sustainability. Enterprises can improve their competitiveness and achieve sustainable growth by improving their performance and operations. Both Shabbir and Wisdom (2020) and Canh et al. (2019) have argued that engaging in profit creation alone limits the sustainable development of enterprises, which can achieve sustainable growth—based on balanced development—only by using CSR and environmental conservation activities to continuously increase their value. According to Bhattarai et al. (2019), researchers can divide SE performance into economic and social performance and analyze the factors that enable SEs to provide social services, such as job creation for vulnerable groups and community development. Baek and Cho’s (2020) analysis of the impact of internal auditor characteristics on management performance and sustainability management have uncovered a relatively negative perception of enterprise performance and sustainability management. Active efforts are therefore needed to manage activities sustainably. Based on previous studies, we propose the following hypotheses:

Hypothesis 3: Social performance has a positive influence on sustainability.
Hypothesis 4: Economic performance has a positive influence on sustainability.

### Mediating Effects of Performance

Corporate social responsibility is a management strategy that strengthens enterprise competitiveness in the long term, generating economic benefits for sustainable growth, and using environmental and social responsibilities strategically to create a competitive advantage (McAdam and Leonard, 2003). CSR improves the lives of local residents by eliminating conflicts and distrust among community stakeholders and increasing local employment and profits, social and economic achievements, and sustainable growth (Hong and Chao, 2018). Enterprises thus use social services, such as CSR, to enhance their performance and to become competitive and sustainable entities. Based on this relationship, we have formulated the following hypotheses:

Hypothesis 5: Social performance has a positive mediating effect on CSR and sustainability.
Hypothesis 6: Economic performance has a positive mediating effect on CSR and sustainability.

### Moderating Effects of Innovativeness

In a challenging environment for enterprise survival, enterprises that cannot respond to new opportunities or technological change will inevitably suffer economic losses. To remain profitable, they must embrace innovation (Lee et al., 2020). Innovative organizations outpace less innovative ones in product and service composition. They are highly competitive and sustainable and pursue sustainable management activities that improve their economic and social performance (Cohen and Levinthal, 1990). Therefore, this study proposes the following hypotheses:

Hypothesis 7: Sustainability moderates the relationship between CSR and social performance.
Hypothesis 8: Sustainability moderates the relationship between CSR and economic performance.

## Research Methodology

### Research Model

**FIGURE 1:**
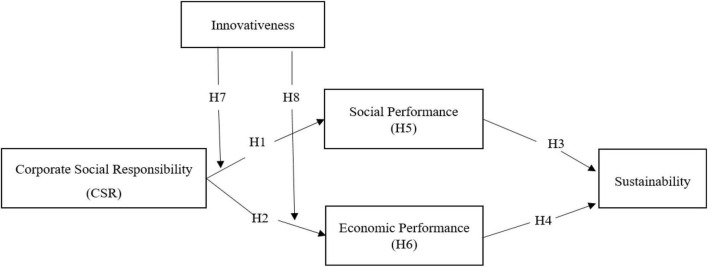
Research model.

Figure 1 presents the research model used in this study. The SPSS 25.0 and AMOS 22.0 programs have been used to verify the hypotheses, in accordance with the research model.

### Sample and Data Collection

The present study examines the effects of CSR on SE sustainability and the mediating and moderating effects of performance and innovativeness on this relationship. To provide data, we have surveyed staff members of SEs distributed across Gwangju and Jeonnam provinces in South Korea. In Korea, there is significant public interest in social responsibility, environmental problems, and sustainable development; as a result, SEs are continuously being established. These SEs are linked to employment policies at the national level because they provide beneficial social services for poor people. However, their dependence on excessive information subsidies hinders their sustainable development. The present study therefore focuses on the sustainability of Korean SEs.

Participants gave their informed consent for their data to be used in this research. The survey was conducted in June 1–30, 2021. Overall, 300 questionnaires were distributed and 274 responses were collected; of these, 226 questionnaires were complete and used in this analysis. The demographic characteristics of the participants were as follows: 137 (60.6%) were men and 89 (39.4%) were women. In terms of age, 38 (16.8%) were under 30; 84 (37.2%) were 31–40; 91 (40.3%) were 41–50; and 13 (5.7%) were over 50. Most respondents had a college degree or higher (82.3%) and were organization executives or presidents (77.4%). Of the SEs, 81.9% were mixed (providing jobs, social services, and community contributions). The major revenue sources for most these businesses (85.8%) were sales and services.

As there was a risk of common-method bias (CMB) if the respondents responded in a socially desirable manner (Podsakoff et al., 2003), the following measures were taken to minimize this limitation: the questionnaire content was laid out clearly and concisely, with items arranged differently. To account for CMB, we confirmed the common variance and cumulative variance values through an exploratory factor analysis. The first factor involved the common and cumulative variance, which were 20.404 and 20.404, respectively. There was no problem with the unification method because the first-factor component did not account for more than 50% of the total change in explanatory power (Podsakoff et al., 2003).

### Measures

The CSR questionnaire was revised and developed using questions developed by Gürlek et al. (2017), which were assessed using four items. Sample items included “This enterprise practices ethical management,” and “This enterprise is actively engaged in social contribution activities.”

Based on Pinheiro et al.’s (2021) research on SE performance measurement, four items were used to measure social performance and five to measure economic performance. Social performance was defined as “performance that includes the mission, service spirit, and social contribution that SEs essentially pursue, such as contributions to the stabilization of society,” while economic performance was defined as “performance that enterprises essentially pursue, such as continuous sales and operating profit, achieving profit targets, and improving customer satisfaction with products” (Bhattarai et al., 2019; Cheah et al., 2019; Doh, 2020).

Sustainability was divided into five measurement items, based on Leung et al. (2019): continuous employment growth in SEs, social-service provision, relationships with government agencies, improved competitiveness, and increased sales.

Innovativeness was defined as “the speed to accept and spread new ideas, opinions, and products before others,” based on Hurt et al. (1977) and Rogers (1995). The measurement tools used were developed by Delmas and Pekovic (2018). All of the items in this study were measured using a 7-point Likert scale (1 = strongly disagree, 7 = strongly agree).

## Results

Table 1 shows the results of the exploratory factor analysis. The results were divided into five independent factors. The items associated with each measurement variable were examined in detail: CSR, EP, and INNO included four items each, while SP and SUS included five items. Cronbach’s alpha was used to test reliability. The results of the reliability test were as follows: CSR = 0.929, EP = 0.953, SP = 0.980, INNO = 0.961, and SUS = 0.981. All of the values of the Cronbach’s alpha coefficient were over 0.7 and the results were confirmed to be highly reliable.

**TABLE 1:** Results of the exploratory factor analysis.

Items	Varimax rotation loadings (*n* = 226)				
	
	SP	SUS	EP	CSR	INNO
SP2	0.946	0.142	0.104	0.056	0.099
SP5	0.944	0.137	0.103	0.047	0.147
SP4	0.939	0.131	0.129	0.048	0.104
SP3	0.935	0.159	0.079	0.057	0.152
SP1	0.926	0.123	0.155	0.073	0.043
SUS5	0.124	0.959	0.059	0.000	0.050
SUS1	0.138	0.948	0.066	0.018	0.095
SUS4	0.159	0.947	0.072	−0.019	0.062
SUS3	0.108	0.943	0.084	−0.006	0.098
SUS2	0.142	0.942	0.087	0.013	0.099
EP2	0.136	0.087	0.885	0.143	0.254
EP4	0.158	0.105	0.847	0.106	0.349
EP3	0.120	0.079	0.836	0.114	0.399
EP1	0.167	0.103	0.789	0.121	0.417
CSR2	0.078	0.012	0.128	0.923	0.113
CSR4	0.058	0.008	0.120	0.913	0.131
CSR1	0.007	−0.011	0.022	0.903	0.144
CSR3	0.085	−0.010	0.133	0.838	0.101
INNO2	0.123	0.101	0.350	0.195	0.858
INNO4	0.147	0.132	0.376	0.162	0.840
INNO1	0.156	0.132	0.456	0.136	0.798
INNO3	0.176	0.100	0.405	0.194	0.782
Eigenvalue	4.685	4.678	3.601	3.398	3.373
Percent explained variance	21.294	21.263	16.370	15.447	15.332
KMO = 0.890 (sig = 0.000)					

*Loadings on items are shaded dark gray. CSR, corporate social responsibility; EP, economic performance; SP, social performance; SUS, sustainability; INNO, innovativeness.*

Table 2 shows the results of the confirmatory factor analysis. The model fit showed *X*^2^(196) = 547.153, *p* < 0.001, *X*^2^/df = 2.792, RMSEA = 0.089, IFI = 0.949, CFI = 0.949, and TLI = 0.940, indicating a satisfactory fit. An RMSEA value under.10 is considered reasonable (MacCallum et al., 1996; Davis et al., 2006). We also checked the convergent validity of the constructs by examining the average variance extraction (AVE) of each construct tested in this study. All of the AVEs were higher than the 0.5 threshold for all constructs (ranging from 0.666 to 0.815), demonstrating convergent validity (Fornell and Larcker, 1981). When the composite reliability (C.R.) values were calculated, CSR = 0.787, EP = 0.881, SP = 0.923, INNO = 0.907, and SUS = 0.924. Since all of the CR values were over 0.70, the measurement tool used in this study was found to be valid. Based on the AVE and CR results, these values were considered significant and acceptable.

**TABLE 2:** Results of the reliability and validity testing.

Variable		SE	CR	*P*	Standardized factor loadings	AVE	CR
CSR	CSR4				0.787	0.666	0.787
	CSR3	0.089	12.752	***	0.710		
	CSR2	0.062	20.267	***	0.927		
	CSR1	0.075	16.469	***	0.826		
EP	EP1				0.844	0.752	0.881
	EP2	0.063	17.564	***	0.836		
	EP3	0.055	22.091	***	0.923		
	EP4	0.06	18.912	***	0.864		
SP	SP1	0.063	18.906	***	0.856	0.797	0.923
	SP2				0.828		
	SP3	0.05	23.715	***	0.936		
	SP4	0.045	24.602	***	0.882		
	SP5	0.047	25.217	***	0.956		
SUS	SUS1				0.816	0.806	0.924
	SUS2	0.047	24.778	***	0.892		
	SUS3	0.061	20.606	***	0.898		
	SUS4	0.047	25.167	***	0.910		
	SUS5	0.059	23.166	***	0.966		
INNO	INNO4				0.904	0.815	0.907
	INNO3	0.048	19.386	***	0.873		
	INNO2	0.037	26.246	***	0.901		
	INNO1	0.047	22.358	***	0.932		

Model fit		*X*^2^(*196*) = 547.153, *p* < 0.001, *X*^2^/df = 2.792, RMSEA = 0.089,					
		IFI = 0.949, CFI = 0.949, TLI = 0.940					

N = 226. CSR, corporate social responsibility; EP, economic performance; SP, social performance; SUS, sustainability; INNO, innovativeness ***p < 0.001, **p < 0.01, *p < 0.05.

Table presents the descriptive statistics. The results of the mean showed that CSR = 0.4.733, EP = 4.920, SP = 4.805, INNO = 5.172, and SUS = 5.000. According to the standard deviation results, CSR = 1.557, EP = 1.412, SP = 1.682, INNO = 1.436, and SUS = 1.170. To verify the discriminant validity between each factor, the square root of AVE was used after a confirmatory factor analysis, based on Fornell and Larcker (1981). Not only was the AVE square root value greater than 0.5, it was also greater than all of the values in the rows and columns, verifying the validity of discrimination among the concepts of composition (Table 3).

**TABLE 3:** Descriptive statistics, reliability, and correlation analysis.

	Mean	SD	1	2	3	4	5
1	4.733	1.557	(0.929)				
2	5.172	1.436	0.350***	(0.961)			
3	4.920	1.412	0.286***	0.763***	(0.953)		
4	4.805	1.682	0.146*	0.330***	0.322***	(0.980)	
5	5.000	1.710	0.029	0.248***	0.220**	0.295***	(0.981)

N = 226; 1 = CSR; 2 = INNO; 3 = EP; 4 = SP; 5 = SUS; CSR, corporate social responsibility; EP, economic performance; SP, social performance; SUS, sustainability; INNO, innovativeness ***p < 0.001, **p < 0.01, *p < 0.05. The diagonal () is the square root of the AVE of each variable.

We conducted a path analysis using the AMOS 22.0 program to test the study hypotheses. Table 4 presents the results of the path analysis. First, the model fit showed *X*^2^ (62) = 103.641, *p* < 0.001, *X*^2^/df = 1.672, RMSEA = 0.055, IFI = 0.989, CFI = 0.989, and TLI = 0.986. According to the results of the path analysis (CSR → EP→ SUS), CSR had a positive influence on EP (estimate = 0.258, *p* < 0.001). In addition, EP had a positive influence on SUS (estimate = 0.293, *p* < 0.001). The indirect effect had an estimated value of 0.069. According to the bootstrap results, the lower and upper bounds were 0.034 and 0.124, respectively. Therefore, the mediating effect of EP was significant.

**TABLE 4:** Path analysis (CSR → EP→ SUS).

Path			Estimate	SE	CR	*p*
CSR	→	EP	0.258	0.058	4.418	***
EP	→	SUS	0.293	0.086	3.386	***
**Mediating effect**			**Indirect effect**	**Lower bounds**	**Upper bounds**	
SCR → EP→ SUS			0.069	0.034	0.124	

Model fit			*X*^2^(*62*) = 103.641, *p* < 0.001 *X*^2^/df = 1.672, RMSEA = 0.055, IFI = 0.989, CFI = 0.989, TLI = 0.986			

N = 226; CSR, corporate social responsibility; EP, economic performance; SP, social performance; SUS, sustainability; INNO, innovativeness ***p < 0.001, **p < 0.01, *p < 0.05.

Next, the model fit showed *X*^2^(71) = 117.560, *p* < 0.001, *X*^2^/df = 1.656, RMSEA = 0.054, IFI = 0.990, CFI = 0.990, and TLI = 0.987. According to the results of the path analysis (CSR →SP→ SUS; see Table 5), CSR had a positive influence on SP (estimate = 0.218, *p* < 0.1). In addition, SP had a positive influence on SUS (estimate = 0.288, *p* < 0.001). The indirect effect had an estimated value of 0.037. According to the bootstrap results, the lower and upper bounds were 0.010 and 0.080, respectively. Therefore, the mediating effect of SP was significant.

**TABLE 5:** Path analysis (CSR → SP→ SUS).

Path			Estimate	SE	CR	*P*
CSR	→	SP	0.218	0.121	1.805	0.071
SP	→	SUS	0.288	0.064	4.526	***
**Mediating effect**			**Indirect effect**	**Lower bounds**	**Upper bounds**	
SCR → SP→ SUS			0.037	0.010	0.080	

Model fit			*X*^2^(*71*) = 117.560, *p* < 0.001, *X*^2^/df = 1.656, RMSEA = 0.054, IFI = 0.990, CFI = 0.990, TLI = 0.987			

N = 226. CSR, corporate social responsibility; EP, economic performance; SP, social performance; SUS, sustainability; INNO, innovativeness ***p < 0.001, **p < 0.01, *p < 0.05.

We used Baron and Kenny’s (1986) moderating effect verification method with SPSS. Hypothesis 7 states that INNO positively moderates the relationship between CSR and EP. To test the moderating effect of EP, we conducted a multiple regression analysis using the SPSS 18. Table 6 presents the moderating effects of INNO on CSR and EP. Model 1 shows that CSR positively influenced EP (β = 0.286, *p* < 0.001). Model 2 shows that INNO had a positive influence on EP (β = 0.756, *p* < 0.001). The moderating effect of INNO was β = −0.114, *p* < 0.001. However, INNO had a negative moderating effect. Therefore, Hypothesis 7 was rejected.

**TABLE 6:** Moderating effect of INNO between CSR and EP.

Dependent: EP							

	Model 1		Model 2		Model 3		VIF
			
	β	*T*	β	*t*	β	*t*	
CSR (A)	0.286***	4.467	0.022	0.472	0.025	0.548	1.140
INNO (B)			0.756***	16.380	0.723***	15.275	1.229
Interaction (A X B)					−0.114*	−2.567	1.084
*R*^2^ (Adjusted *R*^2^)	0.082 (0.078)		0.583 (0.583)		0.595 (0.595)		
△ *R*^2^ (△Adjusted *R*^2^)			0.501 (0.505)		0.012 (0.012)		
*F*	19.951***		156.035***		108.826***		
***p < 0.001, **p < 0.01, *p < 0.05							

N = 226. CSR, corporate social responsibility; EP, economic performance; SP, social performance; SUS, sustainability; INNO, innovativeness ***p < 0.001, **p < 0.01, *p < 0.05.

Hypothesis 8 states that INNO positively moderates the relationship between CSR and SP. Table 7 shows the moderating effects of INNO on CSR and SP. Model 1 shows that CSR positively influences SP (β = 0.146, *p* < 0.05). Model 2 shows that INNO has a positive influence on SP (β = 0.318, *p* < 0.001). Finally, the moderating effect of INNO was β = −0.066, *p* > 0.1. However, INNO had an insignificant moderating effect. Therefore, Hypothesis 8 was rejected.

**TABLE 7:** Moderating effect of INNO between CSR and SP.

Dependent: SP							

	Model 1		Model 2		Model 3		VIF
			
	β	*t*	β	*t*	β	*t*	
CSR (A)	0.146*	2.213	0.035	0.521	0.037	0.549	1.140
INNO (B)			0.318***	4.715	0.299***	4.271	1.229
Interaction (A X B)					−0.066	−0.999	1.084
*R*^2^ (Adjusted *R*^2^)	0.021 (0.017)		0.110 (0.102)		0.114 (0.102)		
△ *R*^2^ (△Adjusted *R*^2^)			0.089 (0.085)		0.004 (0.000)		
*F*	4.898*		13.798***		9.531***		
***p < 0.001, **p < 0.01, *p < 0.05							

N = 226. CSR, corporate social responsibility; EP, economic performance; SP, social performance; SUS, sustainability; INNO, innovativeness ***p < 0.001, **p < 0.01, *p < 0.05.

## Discussion and Conclusion

Given global awareness of social issues, businesses are pursuing sustainable growth and higher performance through activities related to CSR. CSR is vital to the sustainable growth of SEs. SEs that rely heavily on government subsidies can achieve sustainable growth through better economic and social performance by creatively managing innovations to adapt to a rapidly changing environment. This study investigates the effect of CSR on sustainability in SEs, the mediating effect of performance, and the moderating effect of innovation on this relationship. The results of this study suggest that economic and social performance mediate the effect of CSR on the sustainability of SEs. However, innovation had no moderating effect on the relationship between CSR and performance. This suggests that SEs are less willing to embrace new changes or to attempt creative technological innovations. In the future, it will be necessary to help SEs recognize the importance of innovation through technical support. The present study emphasizes the positive advantages of CSR on SE, highlighting the role of performance, which ultimately strengthens the effect of CSR on sustainable development. Despite its limitations, this research provides invaluable insights that can help SEs understand the mechanisms that increase sustainability through CSR activities.

Although many studies have examined the effects of CSR activities on corporate image (Abdulaziz-Alhumaidan and Ahmad, 2019; Zhu et al., 2019), relatively few have explored the way in which CSR affects corporate sustainability through performance. The present study makes a meaningful contribution by empirically verifying sustainability through SE performance. CSR plays a crucial role in enhancing corporate performance (Hernández et al., 2020). Companies can achieve sustainability through performance (Bhattarai et al., 2019; Canh et al., 2019). In previous studies, CSR has been explained as a factor that strongly influences corporate performance (Igwe et al., 2018; Cho and Lee, 2019; Moon and Parc, 2019) and can be used as a powerful variable to improve corporate competitiveness. It is therefore clear that competitiveness ultimately enables sustainability. Digital innovation is rapidly changing the management environment of SEs. In this context, the present study makes an important theoretical contribution, arguing that innovation can improve SE performance. Although this study shows that innovation does not have a moderating effect on improving SE performance, it is essential to make innovative changes in SEs in the future.

Against this backdrop, our empirical analysis of the impact of CSR on sustainability in SEs has identified the mediating effect of performance and the moderating effect of innovativeness. The findings and implications can be summarized as follows. First, CSR on SE has been shown to improve economic and social performance. By pursuing social and economic values that improve the quality of life of community members, SEs can improve their business performance and the community environment. This suggests that CSR is a key responsibility of SEs, which can improve their corporate image and competitiveness through CS activities, thus strengthening their economic and social performance.

Second, the economic and social performance of SEs can lead to improved sustainability. SEs can achieve sustainable growth only when they also achieve good economic and social performance through business activities and social services. They can gain a competitive advantage by employing efficient management strategies, providing creative and innovative social services, improving performance, and ultimately achieving sustainable growth. Through sustainable growth, SEs can offer employment and social services to vulnerable people, achieve stable profit growth, and secure their own competitiveness.

Third, SE performance mediates the relationship between CSR and sustainability. In other words, improved performance is critical to improving the sustainability of SEs, and CSR can achieve that. For SEs to achieve sustainable growth, they must strengthen their economic and social performance by increasing revenue from products and services, based on strong competitiveness, and doing more to develop communities.

Fourth, innovation does not have a moderating effect on CSR of SE and performance in this study. Since SEs seek social goals that provide employment and social services to marginalized people, they tend to be unaware of creative and innovative operational methods that encourage change. However, given the dynamic and unpredictable nature of the business environment, SEs must actively cooperate with their local communities and improve cooperative ties with community stakeholders through innovation to strengthen their business performance and enable sustainable growth. To this end, diverse educational programs must be offered to members of SEs to help them recognize the importance of innovation.

Fifth, SEs needs to achieve goals in the long term. They should make the continual management through the CSR, such as social service provisions, environmental problem solving, and the generation of financial revenue. Also, SEs can achieve their original purpose through providing sustainable jobs and social services.

## Data Availability Statement

The raw data supporting the conclusions of this article will be made available by the authors, without undue reservation.

## Ethics Statement

The studies involving human participants were reviewed and approved by the Ethics Committee of Gachon University. The participants provided written informed consent before taking part in the study.

## Author Contributions

CQ performed data collection and analysis. SJ contributed to drafting, review, and editing. Both authors contributed to the study conception and design.

## Conflict of Interest

The authors declare that the research was conducted in the absence of any commercial or financial relationships that could be construed as a potential conflict of interest.

## Publisher’s Note

All claims expressed in this article are solely those of the authors and do not necessarily represent those of their affiliated organizations, or those of the publisher, the editors and the reviewers. Any product that may be evaluated in this article, or claim that may be made by its manufacturer, is not guaranteed or endorsed by the publisher.
